# Post-GWAS Validation of Target Genes Associated with HbF and HbA_2_ Levels

**DOI:** 10.3390/cells13141185

**Published:** 2024-07-12

**Authors:** Cristian Antonio Caria, Valeria Faà, Susanna Porcu, Maria Franca Marongiu, Daniela Poddie, Lucia Perseu, Alessandra Meloni, Simona Vaccargiu, Maria Serafina Ristaldi

**Affiliations:** Istituto di Ricerca Genetica e Biomedica, Cittadella Universitaria di Monserrato, SS 554, Bivio Sestu Km 4,500, 09042 Cagliari, Italy; cristian.caria@irgb.cnr.it (C.A.C.); valeria.faa@cnr.it (V.F.); susanna.porcu@cnr.it (S.P.); mariafranca.marongiu@irgb.cnr.it (M.F.M.); daniela.poddie@irgb.cnr.it (D.P.); lucia.perseu@irgb.cnr.it (L.P.); alessandra.meloni@irgb.cnr.it (A.M.); simona.vaccargiu@irgb.cnr.it (S.V.)

**Keywords:** beta-hemoglobinopathies, *CCND3*, *NFIX*

## Abstract

Genome-Wide Association Studies (GWASs) have identified a huge number of variants associated with different traits. However, their validation through in vitro and in vivo studies often lags well behind their identification. For variants associated with traits or diseases of biomedical interest, this gap delays the development of possible therapies. This issue also impacts beta-hemoglobinopathies, such as beta-thalassemia and sickle cell disease (SCD). The definitive cures for these diseases are currently bone marrow transplantation and gene therapy. However, limitations regarding their effective use restrict their worldwide application. Great efforts have been made to identify whether modulators of fetal hemoglobin (HbF) and, to a lesser extent, hemoglobin A2 (HbA_2_) are possible therapeutic targets. Herein, we performed the post-GWAS in vivo validation of two genes, cyclin D3 (*CCND3*) and nuclear factor I X (*NFIX*), previously associated with HbF and HbA_2_ levels. The absence of *Ccnd3* expression in vivo significantly increased g (HbF) and d (HbA_2_) globin gene expression. Our data suggest that CCND3 is a possible therapeutic target in sickle cell disease. We also confirmed the association of *Nfix* with γ-globin gene expression and present data suggesting a possible role for Nfix in regulating Kruppel-like transcription factor 1 (*Klf1*), a master regulator of hemoglobin switching. This study contributes to filling the gap between GWAS variant identification and target validation for beta-hemoglobinopathies.

## 1. Introduction

More than 15 years ago, the Wellcome Trust Case Control Consortium published the first genome-wide association study (GWAS) of ~14,000 cases of seven common diseases and ~3000 shared controls [1]. Since then, an impressive number of variants have been associated with different traits, pathophysiological states, or disorders affecting thousands of individuals worldwide [2,3,4], establishing GWAS as a milestone in genetic research. Variants in non-coding regions that affect gene expression have increased our knowledge of the complex mechanisms of gene regulation and their implications in disease development. While this approach has demonstrated its key role in identifying new susceptibility genes for diseases and traits, the functional validation of the observed association is often lacking [5,6]. Moreover, the causative variant/gene in an associated region often remains elusive due to linkage disequilibrium. Nonetheless, understanding the underlying biological process is pivotal for translating these findings into clinical care.

This issue also concerns beta-hemoglobinopathies, such as beta-thalassemia (MIM: 613985) and sickle cell disease [MIM: 603903], for which definitive therapies, such as bone marrow transplantation or gene therapy, are extremely limited in their global application. Beta-hemoglobinopathies constitute the most common monogenic disorders, afflicting hundreds of thousands of individuals worldwide [7,8,9,10,11]. Therefore, the global burden of hemoglobinopathies is very high and expected to increase in the coming years [10]. While it may represent the future of clinical medicine, most patients worldwide cannot currently benefit from gene therapy. One of its major obstacles is its cost per patient [12], limiting its use not only in low-income countries, but also in high-income countries. Therefore, intervening with an affordable drug available to almost all patients would be highly desirable. Consequently, identifying and validating genes as candidates for drug treatment against these disorders is currently an important goal of genetic research. To date, the most broadly used US Food and Drug Administration-approved drug for treating adults with SCD is hydroxyurea (HU), a known fetal hemoglobin (HbF) inducer that reduces both the polymerization of sickle cell hemoglobin (HbS) and pain but has many limitations. The HbF levels reached with HU treatment are also not clinically relevant in beta-thalassemia patients [13].

Great effort has been invested in identifying modulators of HbF and, to a lesser extent, hemoglobin A_2_ (HbA_2_) [14,15,16,17,18,19,20]. Amelioration of the beta-thalassemia or sickle cell phenotype by increasing HbF levels has been extensively proven, and key regulators of γ-globin gene (hemoglobin subunit gamma 1/2 [*HBG1/2* [MIM: 142200/142250]) expression, such as BCL11 transcription factor A (BCL11A) or zinc finger and BTB domain-containing 7A (ZBTB7A/LRF), have been identified [14,15,16,17,21]. Less studied is the possibility of modulating HbA_2_, which is expressed, in adult life, at low levels but is fully functional [22,23,24]. However, the previous results of preclinical disease models from our group [18,19] and others [20,25,26,27] have validated HbA_2_ as a therapeutic target for beta-hemoglobinopathies.

A recent GWAS identified two genes: cyclin D3 (*CCND3* [MIM: 123834]) and nuclear factor I X (*NFIX* [MIM: 124005]) [28]. *CCND3* has been associated with HbF and HbA_2_ levels, while *NFIX* has been associated with HbF levels [28]. CCND3 belongs to the cyclin family of proteins, which exert essential functions in cell cycle regulation. The complex formation of CCND3 or the other two Cyclin D1/2 (CCND1/2) with cyclin-dependent kinases 4 (CDK4) and 6 (CDK6) causes the phosphorylation of Retinoblastoma protein (pRB) and its related proteins, which, in turn, release E2F transcription factor 2 (E2F2), a key transcription factor that drives the transition from the G1 to S phase of the cell cycle [29,30,31]. In humans, variants mapped in an erythroid-specific enhancer found upstream of the *CCND3* promoter have been associated with a decrease in the red blood cell (RBC) count and an increase in the mean corpuscular volume (MCV) [32,33]. Since erythropoiesis is tightly connected to the cell cycle, *CCND3* deprivation in mice, although not hampering the complete maturation of the erythroblasts, affects their final size and count. *CCND3*-null mice are viable and fertile and do not show important signs of anemia [32]. Moreover, CCND3 is a druggable target since a clinically approved CDK4/6 inhibitor is currently used to treat breast and other type of cancers [34,35,36].

NFIX belongs to the nuclear factor one (NFI) family of DNA-binding proteins (NFIA, NFIB, NFIC, NFIX), which have been identified as important transcription factors [37]. *NFIX* expression and activity have been detected in multiple tissues and seem to participate in various organ functions and development [38,39]. *NFIX* is essential for brain development, and *Nfix* knockout mice show bone abnormalities [38], limited perinatal or postnatal viability, and impaired feeding with subsequent reduced growth [40,41]. Variants or microdeletions in the *NFIX* gene have also been associated with multiple human disorders, including Malan syndrome (MALNS [MIM: 614753]) and Marshal-Smith syndrome (MRSHSS [MIM: 602535]) [42,43].

The objective of our study was to experimentally evaluate, in a validated transgenic mouse model containing the full human beta-globin cluster [44], the potential of CCND3 and NFIX as modulators of HbF and HbA2.

The results we present in this work confirmed, in vivo, the associations reported earlier [28] and suggest CCND3 as a possible therapeutic target for beta-hemoglobinopathies. 

## 2. Materials and Methods

### 2.1. Mice

The original ln72 [44] (provided by Dr. Frank Grosveld’s laboratory) *Nfix* mouse line was provided by Dr. Graziella Messina (Dipartimento di Bioscienze, Università degli Studi di Milano, Milan, Italy) and Dr. Richard M. Gronostajski (State University of New York at Buffalo, USA) (Aut. n. 748/2015-PR del 22/07/2015). The *Ccnd3* mouse line was kindly *pr*ovided by Dr. Piotr Sicinski (Department of Cancer Biology, Dana-Farber Cancer Institute, Harvard Medical School, Boston, MA, USA). All mouse lines were maintained on a hybrid C57BL6/CBA/J background. All procedures conducted on the mice were in accordance with the rules and regulations set by the Ethical Committee (OPBA) of the University of Cagliari and authorized by the Ministero della Salute (Authorization No.: 633/2023-PR). Genotypes were assigned by polymerase chain reaction (PCR) from genomic DNA according to The Jackson Laboratory protocols (https://www.jax.org/jax-mice-and-services/customer-support/technical-support/genotyping-resources accessed on 8 March 2023). The gene name and sequence of primers used for genotyping are listed in Table S1. 

### 2.2. Real-Time 

Real-time quantitative PCR (RT-qPCR) was performed on RNA extracted from hematopoietic tissues (fetal liver and bone marrow) in different developmental growth phases and from the HEL cell line. Total RNA was extracted using TRIZOL LS (Thermo Fisher Scientific, Invitrogen, Waltham, MA, USA) according to the manufacturer’s instructions, treated with DNase I (Thermo Fisher Scientific, Invitrogen, Waltham, MA, USA) and cDNA retro-transcribed using Superscript III reverse transcriptase (Thermo Fisher Scientific, Invitrogen, Waltham, MA, USA). RT-qPCRs were performed using SYBR Green chemistry (Thermo Fisher Scientific, Applied Biosystems, Waltham, MA, USA) with an ABI PRISM 7900 thermocycler (Thermo Fisher Scientific, Applied Biosystems, Waltham, MA, USA). The reactions were performed on at least three different samples in triplicate. The expression of fetal and adult globins was calculated by the 2^−ΔΔCt^ method [45], using mouse or human *α-globin* mRNA as the reference control. The gene name and sequence of primers used for RT-qPCR are listed in Table S1.

### 2.3. Hematology

Hematological analyses were conducted by collecting 0.2 mL of blood by cardiac puncture from previously sacrificed adult mice. The blood was collected in Microtainer ethylenediamine tetra acetic acid (EDTA) collection tubes. The analyses were performed using HemoCue Hemoglobin and Automated Hematology Cell Counter MS4 (Melet Schloesing Laboratories, Osny, France). At least three different animals were used for the analyses.

### 2.4. Flow Cytometry

Analyses were conducted on freshly isolated cells (1 × 10^5^ each sample) from adult bone marrow, from any group, and analyzed, as previously described [46], according to the levels of expression of anti-mouse Ter119 fluorescein isothiocyanate (FITC) and anti-mouse CD71 phycoerythrin (PE) antibodies (BD-Bioscience, San Jose, CA, USA) at a final concentration of 1:100. The Klf1 expression was analyzed after the fixation and permeabilization of the cells using the Cytofix/Cytoperm Fixation/Permabilization Kit (BD Bioscience, San Jose, CA, USA). Briefly, 100 μL of fixation/permeabilization solution was added to the cells (1 × 10^5^ cells each sample, 20 min at 4 °C) and washed with 1 × BD Perm/Wash buffer. Labeling of the cells was performed by using rabbit anti-mouse anti-Klf1 (LSBio, LifeSpan Bioscience, Lynnwood, WA, USA) at a final concentration of 1:100, and secondary antibody Goat anti-rabbit Alexa Fluor 488 (Thermo Fisher Scientific, Invitrogen, Waltham, MA, USA) at a final concentration of 1:400. The cells were incubated for 20 min at 4 °C (dark room), washed in phosphate-buffered saline (5% bovine serum albumin), and re-suspended in fluorescence-activated cell sorting (FACS) flow solution (BD-Bioscience, San Jose, CA, USA). The secondary antibody was incubated for 20 min at room temperature (dark room). Data were recorded using a FACSCanto cytometer (BD Bioscience, San Jose, CA, USA) and analyzed by FACSDiva software Version 6.1.3 (BD Bioscience, San Jose, CA, USA) and Flowjo v7.6.5 (BD-Bioscience, San Jose, CA, USA). Each analysis was conducted on at least three mice. 

### 2.5. Constructs

The NFIX cDNA obtained by the K562 cell line was amplified by PCR using primers that carry EcoRI and XbaI restriction sites. The fragment was then cloned into a pEF5HA vector that allows the expression of proteins (Tag/fusion protein HA at N-terminal) under the control of the EFalpha promoter. A portion of the *KLF1* promoter (from −516 to −1 from the transcription start site) was amplified from genomic DNA using primers that carry BglII/HindIII restriction sites and cloned into the pGL4.70 (hRLUC) vector (PROMEGA, Madison, WI, USA) (Table S1). 

### 2.6. Cell Culture and Transient Transfections

HEL 92.1.7 (erythroblast cell line isolated from bone marrow of patient with erythroleukemia) and human erythroleukemic K562 cells were cultured in RPMI-1640 medium supplemented with 10% FBS (fetal bovine serum) (Euroclone, Pero, Milano, Italy), 1% penicillin, 1% streptomycin, and 1% L-glutamine (Thermo Fisher Scientific, Invitrogen, Waltham, MA, USA) in a humidified 5% CO_2_ atmosphere at 37 °C. Transfections were performed using Lipofectamine LTX reagent according to the manufacturer’s instructions (Thermo Fisher Scientific, Invitrogen, Waltham, MA, USA). Hela cells (epithelial cells isolated from the human cervix of a patient with adenocarcinoma) were cultured in Dulbecco’s modified Eagle’s minimal essential medium (DMEM) supplemented with 10% FBS, 1% penicillin, 1% streptomycin, and 1% L-glutamine (Thermo Fisher Scientific, Invitrogen, Waltham, MA, USA) in a humidified 5% CO_2_ atmosphere at 37 °C. Transfections were performed using Lipofectamine 2000 Transfection reagent (Thermo Fisher Scientific, Invitrogen, Waltham, MA, USA) according to the manufacturer’s instructions. The HEL 92.1.7, K562, HeLa cells were originally obtained from ATCC (American Type Culture Collection, Manassas, VA, USA).

### 2.7. Luciferase Assay

Here, 1 ug of pGL4- KLF1 promoter alone or together with 0.5 ug of pEF5HA- NFIX vector was transfected into Hela cells. After incubation for 48 h, the cells were washed with 1 × PBS buffer and lysed. Luciferase assays were performed on cell lysates using the Dual-Luciferase reporter assay kit from PROMEGA (Madison, WI, USA) according to the manufacturer’s instructions and by reading the luminescence on a Synergy 2 luminometer (BIOTEK, Agilent, Santa Clara, CA, USA). Each experiment was performed three times on a triplicate sample. 

### 2.8. Statistical Analysis 

All data presented in this paper were obtained by performing technical and biological triplicates. The statistical significance of the results was calculated using a *t*-test, with *p* < 0.05 considered significant. The error bars of each plot represent one standard deviation from the mean.

## 3. Results

### 3.1. Ccnd3 Deprivation Increases the Expression of γ- and δ-Globin Genes

**Figure 1:**
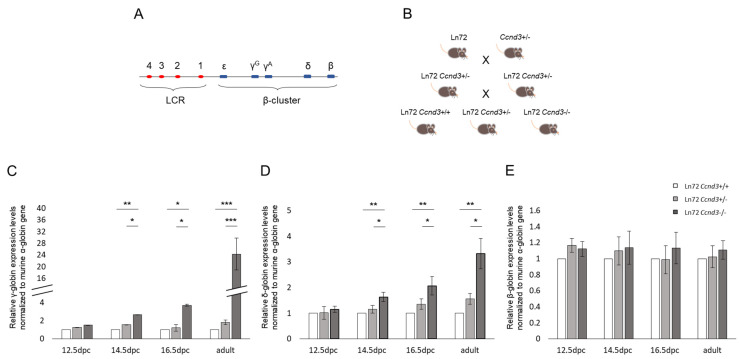
(**A**) Schematic representation of the human β-globin cluster transgene in ln72 mice, comprising four LCR DNAseI hypersensitivity sites and the entire β-globin gene cluster. (**B**) Breeding strategy adopted to obtain ln72 *Ccnd3*+/+, ln72 *Ccnd3*+/− and ln72 *Ccnd3*−/− mice. (**C**) γ-globin, (**D**) δ-globin, and (**E**) β-globin gene expression levels from embryos and adult mice obtained by RT-qPCR. Data are normalized to mouse *α-globin* gene expression and indicated as the mRNA fold change expression relative to the control values (ln72 *Ccnd3*+/+). Each datum is representative of five independent experiments (at least three mice in each experiment). The error bars represent the standard deviation from the mean. (*p*-value: * < 0.05; ** < 0.01; *** < 0.001).

To investigate if and to what extent the absence of Ccnd3 can increase the expression levels of the hemoglobin γ and δ (*HBD* [MIM: 142000]) subunits, we inter-crossed a *Ccnd3*+/− mouse model with a transgenic mouse line carrying the entire human β-globin gene cluster (line 72 [ln72]) (Figure 1A) [44] to obtain ln72 *Ccnd3*−/− and ln72 *Ccnd3*+/− mice and ln72 *Ccnd3*+/+ mice as controls (Figure 1B). The γ-, δ- and β-globin (*HBB* [MIM: 141900]) gene expression was evaluated through RT-qPCR in the fetal liver of embryos at 12.5, 14.5 and 16.5 days post coitum (dpc) and in the bone marrow of adult mice (2–3 months) through quantitative PCR (RT-qPCR); the results were indicated as the fold change expression relative to the control mice values. 

The expression of the three studied globin genes did not differ significantly between ln72 *Ccnd3*+/− and ln72 *Ccnd3*+/+ mice at any of the examined development time points. At 12.5 dpc, the γ-globin gene expression did not differ significantly among the three genotypes (Figure 1C, Table S2). Starting from 14.5 dpc, we observed a progressive and significant increase in the fetal globin mRNA levels in ln72 *Ccnd3*−/− mice compared to the ln72 *Ccnd3*+/+ controls (Figure 1C, Table S2). This increase reached its maximum in adult mice, where the γ-globin levels were 24.33 ± 5.53 (*p* < 0.0001) higher in ln72 *Ccnd3*−/− mice than in the ln72 *Ccnd3*+/+ controls (Figure 1C, Table S2). A similar pattern was observed with δ-globin gene expression. Indeed, we observed that the δ-globin mRNA levels progressively increased from 14.5 dpc to adulthood, peaking in adult mice, where the δ-globin level was 3.33 ± 0.60 (*p* < 0.01) higher in ln72 *Ccnd3*−/− than in ln72 *Ccnd3*+/+ mice (Figure 1D, Table S2). No significant differences were observed in the β-globin mRNA levels among the three groups of animals at any of the examined development time points analyzed (Figure 1E, Table S2). These results indicate that *Ccnd3* deprivation in adult ln72 mice substantially increases the expression of γ- and δ-globin genes. This result was further confirmed by flow cytometry analysis (Figure S3).

### 3.2. Ccnd3 Deprivation Affects Bone Marrow Erythropoiesis

**Figure 2:**
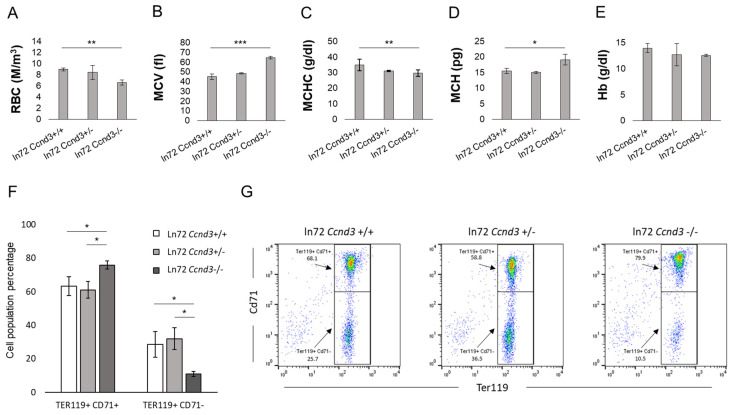
(**A**) RBC, (**B**) MCV, (**C**) MCHC, (**D**) MCH, and (**E**) Hb parameters obtained from adult mice hematological analysis. Each datum is representative of five mice (*n* = 5). (**F**) Percentage of Ter119+ Cd71+ and Ter119+ Cd71−populations from flow cytometry analysis of adult mice of each genotype. Data is representative of five mice (*n* = 5). (**G**) Dot plot of representative erythropoiesis from mice of each genotype and relative percentages. (*p*-value: * < 0.05; ** < 0.01; *** < 0.001).

The hematological analysis of adult ln72 *Ccnd3*+/+, ln72 *Ccnd3*+/−, and ln72 *Ccnd3*−/− mice showed a significant 26.6% decrease in the RBC count (Figure 2A; Table S4) and a significant 30.0% increase in the MCV (Figure 2B and Figure S4, Table S4) in ln72 *Ccnd3*−/− compared to the ln72 *Ccnd3*+/+ controls. Moreover, the mean corpuscular hemoglobin concentration (MCHC) was 14.6% lower, and the mean corpuscular hemoglobin (MCH) was 18.7% higher in the ln72 *Ccnd3*−/− mice than in the controls (Figure 2C,D; Table S4). These data are in agreement with previously reported data for *Ccnd3−*/*−* mice [32]. The total hemoglobin (Hb; Figure 2E, Table S4), hematocrit, and red cell distribution width did not differ significantly among the three groups. 

The impact of *Ccnd3* deprivation on bone marrow erythropoiesis was examined through flow cytometry according to the Ter119/Cd71 expression levels [46]. A significant increment of about 12–13% was found on Ter119+ Cd71+ cells, which are mainly composed of basophil and polychromatic normoblasts, in ln72 *Ccnd3*−/− mice compared to the ln72 *Ccnd3*+/+ controls (Figure 2F,G). In contrast, the Ter119+ Cd71− population (comprising mainly orthochromatic cells) was significantly lower in ln72 *Ccnd3*−/− mice compared to ln72 *Ccnd3*+/+ (by 17.7%) and ln72 *Ccnd3*+/− (by 21%) mice (Figure 2F,G). This delay in erythropoiesis could be caused by the reduction in cell cycle events, resulting in decreased RBCs characterizing the *Ccnd3*−/− phenotype in mice. No significant differences were observed between the ln72 *Ccnd3*+/+ and ln72 *Ccnd3*+/− mice (Figure 2F,G).

### 3.3. Nfix Deprivation Increases the Expression of γ-Globin Gene

**Figure 3:**
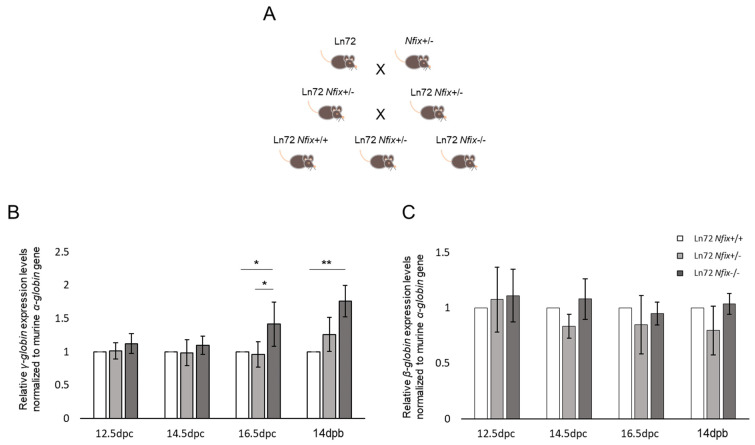
(**A**) Breeding strategy adopted to obtain ln72 *Nfix*+/+, ln72 *Nfix*+/− and ln72 *Nfix*−/− mice. (**B**) γ-globin and (**C**) β-globin genes levels from embryos and adult mice obtained by RT-qPCR. Data are normalized to mouse α-globin and indicated as fold change in mRNA expression relative to control values. Each datum is representative of five independent experiments (at least three mice in each experiment). The error bars represent one standard deviation from the mean. (*p*-value: * < 0.05; ** < 0.01).

To investigate if and to what extent the absence of *Nfix* can increase the expression levels of the hemoglobin γ subunit, we inter-crossed a *Nfix*+/− mouse model with the transgenic mouse ln72 to obtain ln72 *Nfix*−/−, ln72 *Nfix*+/− and ln72 *Nfix*+/+ as control mice (Figure 3A). The γ and β-globin expression was quantified, through RT-qPCR, in fetal liver cells from embryos at the same time points (12.5, 14.5, and 16.5 dpc) as those used for the ln72 *Ccnd3* mice. However, due to the postnatal early lethality of the *Nfix*−/− phenotype in mice, bone marrow was collected from mice at 14 days post birth (dpb). As in ln72 *Ccnd3* mice, the results were indicated as the fold change expression relative to the control mice values. The expression of the studied genes did not differ significantly between the ln72 *Nfix*+/− and ln72 *Nfix*+/+ mice at any of the examined development time points. In addition, the γ-globin expression did not differ significantly among the three genotypes at 12.5 and 14.5 dpc (Figure 3B, Table S3). However, the γ-globin expression was significantly higher in ln72 *Nfix*−/− mice than in the ln72 *Nfix*+/+ controls at 16.5 dpc (+1.41 ± 0.33, *p* < 0.05; Figure 3B, Table S3). A similar result was obtained at 14 dpb, with the γ-globin expression being +1.76 ± 0.24 higher in ln72 *Nfix*−/− mice than in the ln72 *Nfix*+/+ controls (*p* < 0.01; Figure 3B, Table S3). The β-globin expression did not differ significantly among the ln72 *Nfix*−/−, ln72 *Nfi*x+/−, and ln72 *Nfix*+/+ mice (Figure 3C, Table S3).

The hematological and flow cytometry analysis of bone marrow cells showed no significant differences in the red cell parameters between ln72 *Nfix*−/− mice and ln72 *Nfix*+/+ mice (Figures S1 and S2; Table S5). Altogether, these results rule out that NFIX plays a role in erythropoiesis but support its involvement in silencing the γ-globin gene.

### 3.4. Nfix Affects Klf1 Expression

**Figure 4:**
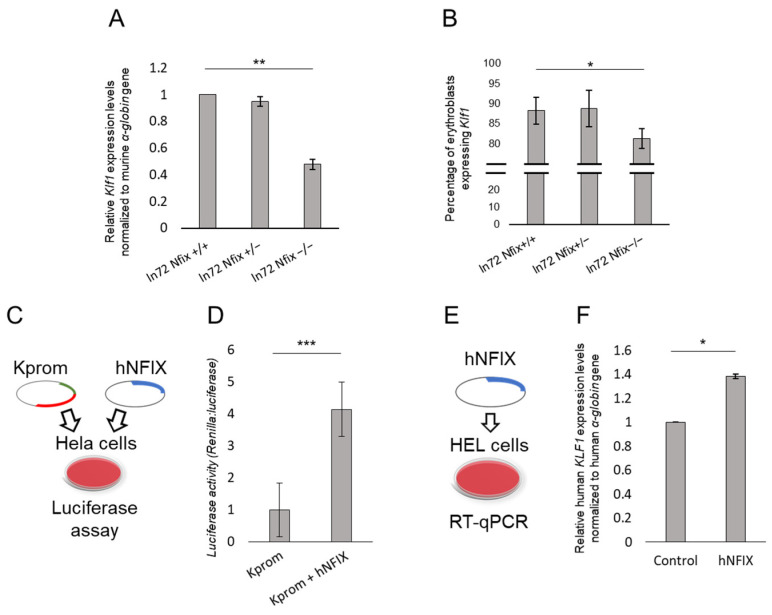
(**A**) Murine *Klf1* expression levels obtained by RT-qPCR of the three ln72 *Nfix* genotypes. (**B**) Percentage of murine Klf1 positive erythroblast cells from flow cytometry analysis of each group of adult mice. (**C**) Schematic representation of luciferase assay. (**D**) Luciferase assay results using the *Klf1* promoter in the absence (Kprom) or presence (Kprom + hNFIX) of the human NFIX protein in Hela cells. Results are normalized to luciferase activity for each sample and values are represented relative to the Kprom construct. (**E**) Schematic representation of transactivation assay. (**F**) Human *KLF1* gene expression level following transactivation with an expression vector (pEF5HA) containing human *NFIX* cDNA in HEL cells. RT-qPCR data are normalized to human α-globin gene expression and indicated as fold change in mRNA expression relative to control values. Each datum is representative of three independent triplicate experiments. The error bars represent one standard deviation from the mean. (*p*-value: * < 0.05; ** < 0.01; *** < 0.001).

A recent study demonstrated that NFIX affects HbF levels, in concert with NFIA, via two mechanisms: the direct repression of the *HBG1/2* genes and an indirect activation of the *BCL11A* gene [47]. The major erythroid-specific activator of *BCL11A* is KLF transcription factor 1 (*KLF1*; [MIM: 600599]), a recognized master regulator of both erythropoiesis and hemoglobin switching [48]. Hemoglobin switching is a highly coordinated process that requires the fine-tuning of several actors. Hence, we wondered whether there was any functional relationship between NFIX and KLF1 since both appear to be involved in activating BCL11A. Therefore, we quantified the *Klf1* expression in bone marrow cells from ln72 *Nfix*−/−, ln72 *Nfix*+/−, and ln72 *Nfix*+/+ mice through RT-qPCR. Surprisingly, the *Klf1* expression was 50% lower in ln72 *Nfix*−/− mice than in the ln72 *Nfix*+/+ controls (Figure 4A). This result was further supported by a flow cytometry analysis of freshly isolated bone marrow cells from the three groups (Figure 4B). The reduced Klf1 expression resulted in an 8% reduction in ln72 *Nfix*−/− mice compared to the controls (Figure 4B). 

To ascertain whether NFIX would directly regulate *KLF1* expression, we performed a dual luciferase assay in HeLa cells using a reporter vector containing part of the human *KLF1* promoter (Kprom) co-transfected with an expression vector containing the human *NFIX* cDNA (Kprom + hNFIX) (Figure 4C). The luciferase activity was fourfold higher with Kprom + hNFIX than with the Kprom control (Figure 4D). To further validate the involvement of NFIX in KLF1 expression, we performed a transactivation assay by transfecting the *NFIX* gene into a human erythroid cell line (HEL) and quantifying the endogenous *KLF1* expression through RT-qPCR (Figure 4E). NFIX overexpression increased the *KLF1* mRNA expression by 40% compared to cells transfected with the empty vector (Figure 4F). Altogether, our data suggest the direct role of Nfix in *Klf1* regulation.

## 4. Discussion

This study aimed to validate, in vivo, two genes implicated by GWAS in the modulation of HbF and HbA_2_ levels: *CCND3* and *NFIX*. The *NFIX* association was restricted to the HbF levels in humans, while *CCND3* was also associated with the HbA_2_ levels [28].

The therapeutic efficacy of HbF in treating SCD and beta-thalassemia is emphasized by the condition associated with the high persistence of fetal hemoglobin (HPFH), a naturally occurring condition in which HbF is expressed in adults at much higher levels than normal. While higher HbA_2_ levels in beta-thalassemia carriers have been described (from a basal level of about 3.0% to 5.8%), there is no clear evidence that variants determine the increase in HbA_2_ at therapeutic levels to date. Nonetheless, we and others have described the validation of HbA_2_ as a therapeutic target for beta-hemoglobinopathies [18,19,23,24]. Unlike HbF, whose expression is almost abolished after birth and restricted to F cells in adults, HbA_2_ has the advantage of being expressed pancellularly in adults. Moreover, the oxygen affinity of HbA_2_ is more similar to HbA1 than HbF [49].

The primary goal of SCD research is to resolve or ameliorate the disease, and this relies on delaying the time of fiber formation during red cell transit through microcirculation, where hypoxia triggers HbS polymerization so that red cells can enter and exit vessels without sickling [50,51,52,53,54,55,56,57]. In healthy humans, HbF and HbA_2_ represent 0.3% and 2.7% of total hemoglobin on average, respectively [28]. If the lack of *CCND3* expression would achieve the same increase in humans as we observed in mice, the HbF and HbA_2_ levels would increase to about 7.3% and 9.0%, respectively, reaching 16.3% of the total hemoglobin. Observations in HU-treated patients and clinical studies have indicated that the positive effects on this condition become noticeable once the HbF level reaches 10% [55,58,59]. While an average HbF level of 30% (10 pg/cell) is considered to completely inhibit sickling [60], a pan-cellular 10% increase in β-like globin is considered sufficient to ameliorate major organ failure, such as stroke or aseptic necrosis [19,58,59]. The anti-sickling properties of HbA_2_ have been described to be similar to those of HbF [19,20,61,62]. Therefore, the projected combined increase in HbF and HbA_2_ (pancellularly expressed) caused by a lack of *CCND3* could improve patients’ conditions.

Of particular interest is the reduction in MCHC observed in *Ccnd3−*/*−* mice. In SCD, the kinetics of fiber formation triggered by hypoxia depend on different factors that make polymerization thermodynamically favorable. One such factor is the hemoglobin concentration within each erythrocyte, with a 10% reduction in MCHC delaying the polymerization time and, thus, producing a therapeutic effect [55]. Therefore, the decrease in MCHC observed in this mouse model could further contribute to ameliorating the clinical symptoms in patients with SCD [55].

However, the observed increases in γ- and δ-globin gene expression could only minimally affect patients with beta-thalassemia since the estimated increase in hemoglobin would only be about two grams per deciliter [28]. Nonetheless, carriers of sickle-cell anemia and beta-thalassemia have, on average, higher HbF and HbA_2_ levels. Therefore, higher HbF and HbA_2_ levels could be expected in carriers and patients due to CCND3 modulation. However, this remains to be experimentally evaluated.

The exact mechanisms by which the perturbation of erythropoiesis, as observed in *Ccnd3*−/− mice, induces changes in globin gene expression are, at present, poorly understood. However, an association between changes in hematopoiesis kinetics with HbF and HbA_2_ levels has been previously proposed [21,63,64,65]. Recently published data from our laboratory have shown that the changes in cell cycle kinetics caused by interferon-β lead to higher levels of δ-globin levels in both mice and humans [49]. Furthermore, it has been described that HU increases HbF levels and F-cell percentages also by blocking the S phase in erythroid progenitor cells, thus perturbing cell cycle progression [66,67]. Both during ontogenesis and adult terminal erythropoiesis, genes in the β-globin cluster are sequentially activated based on their proximity to the locus control region, with more proximal genes expressed at early phases and more distal genes expressed at later phases in differentiation [24,65,68]. These observations could explain, at least in part, the increased expression of γ- and δ-globin genes in *Ccnd3*−/− mice. The lack of *Ccnd3* would lead to the production of mature erythrocytes that maintain higher levels of γ- and δ-globins due to fewer cell divisions, thus shortening the differentiation process.

The existing published data on the possible role of NFIX in γ-globin gene silencing are contradictory. Data from the Blobel lab indicate the combinatorial regulation of HbF by NFIA and NFIX [47]. In their results, the CRISPR-directed downregulation of *NFIX* alone only slightly increased the HBF levels in vitro and in mice xeno-engrafted with human *NFIX*−/− CD34+ cells. Our data in transgenic mice also showed the modest effect of *Nfix* deprivation on γ-globin expression. Conversely, the Shearstone lab recently reported that the short hairpin RNA knockdown of *NIFX* in cord blood- and bone marrow-derived human erythroid cells increased HbF levels to an extent comparable to that caused by downregulating two recognized γ-globin regulators such as BCL11A and LRF [69]. They suggested that the different outcomes after *NFIX* silencing could reflect the different approaches used: CRISPR/Cas9 gene editing versus short hairpin RNA knockdown. Our study is the first attempt to examine the effect of complete *Nfix* silencing in vivo on *HBG1/2* gene regulation. While validating previous GWAS, the magnitude of γ-globin expression increase appears far from being considered beneficial in treating beta-hemoglobinopathies. We have also shown the involvement of Nfix in regulating *Klf1,* a key player in hemoglobin switching.

While our data do not show that Nfix has a robust effect on HbF levels, it is likely that the network of interactions in which Nfix is involved in the regulation of hemoglobin switching is complex and involves other molecular players. This conclusion is also suggested by observations that NFIX may act as an activator of *Klf1* (our data) and *Bcl11a* [47], two of the main players in hemoglobin switching. Further and more in-depth studies, for example using tissue-restricted and/or inducible mouse models, are needed to fully elucidate the role of NFIX in HbF expression.

## 5. Conclusions

Overall, our results validated, in vivo, the previously reported GWAS genetic associations [28] of *CCND3* with HbF and HbA_2_ levels and *NFIX* with HbF levels. Our data also suggest that CCND3 could be considered a potentially valid and druggable therapeutic target for beta-hemoglobinopathies, particularly SCD. Nevertheless, the exact molecular mechanisms underlying the influence of *CCND3* and *NFIX* genes on hemoglobin regulation are not fully elucidated and will require further investigation.

The erythroid phenotype of cyclin D3 is due to its interaction with CDK4 and CDK6. Small-molecule inhibitors of these kinases could be used as a pharmacological approach. At least one CCND3-dependent kinase inhibitor molecule is already in the clinic and could be evaluated in a preclinical model for repositioning [70]. Alternatively, the knockdown of cyclin D3 could be achieved, for example, by developing RNA-based drugs such as siRNA [71].

However, future directions for the potential therapy of beta-hemoglobinopathies based on the modulation of CCND3 expression will require additional studies in vitro and in vivo, using appropriate preclinical models. Success in preclinical disease models could then lead to experimental clinical evaluation.

## Data Availability

Data are available from the corresponding author upon reasonable request.

## Supplementary Materials

The following supporting information can be downloaded at: https://www.mdpi.com/article/10.3390/cells13141185/s1, Figure S1: Hematological parameters from ln72 *Nfix*+/+, ln72 *Nfix*+/− and ln72 *Nfix*−/− mice. Plots show values regarding RBC, MCV, MCHC, MCH and Hb parameters. The error bars represent the standard deviation from the mean; Figure S2: Flow cytometry from adult ln72 *Nfix* mice. Bar plot represents erythropoiesis analysis according to Ter119/Cd71 levels of expression. The error bars represent the standard deviation from the mean; Figure S3: Histograms representing (**A**) γ- and (**B**) δ-globin subunits positive cells in ln72 *Ccnd3*+/+ and ln72 *Ccnd3*−/− mice models; Figure S4: Comparison between erythrocytes volume of ln72 *Ccnd3*+/+ and ln72 *Ccnd3*−/− mice models, left panel represents erythrocytes from ln72 *Ccnd3*+/+ while right panel represents ln72 *Ccnd3*+/+ mice erythorcytes. Bar scale = 5 µm; Table S1: Gene name, sequence and application of primers used in this study; Table S2: data from RT-qPCR on ln72 *Ccnd3* mice; Table S3: data from RT-qPCR on ln72 *Nfix* mice; Table S4: Hematological parameters of ln72 Ccnd3 mice; Table S5: Hematological parameters of ln72 Nfix mice.
