# Supplementary material for: Post-GWAS Validation of Target Genes Associated with HbF and HbA2 Levels
Source: Cells. 2024 Jul 12;13(14):1185. doi: 10.3390/cells13141185 (PMC11274989; doi:10.3390/cells13141185)
Supplement: Supplementary file 1 [file cells-13-01185-s001.zip › cells-3045215-supplementary.pdf]

Supplementary materials

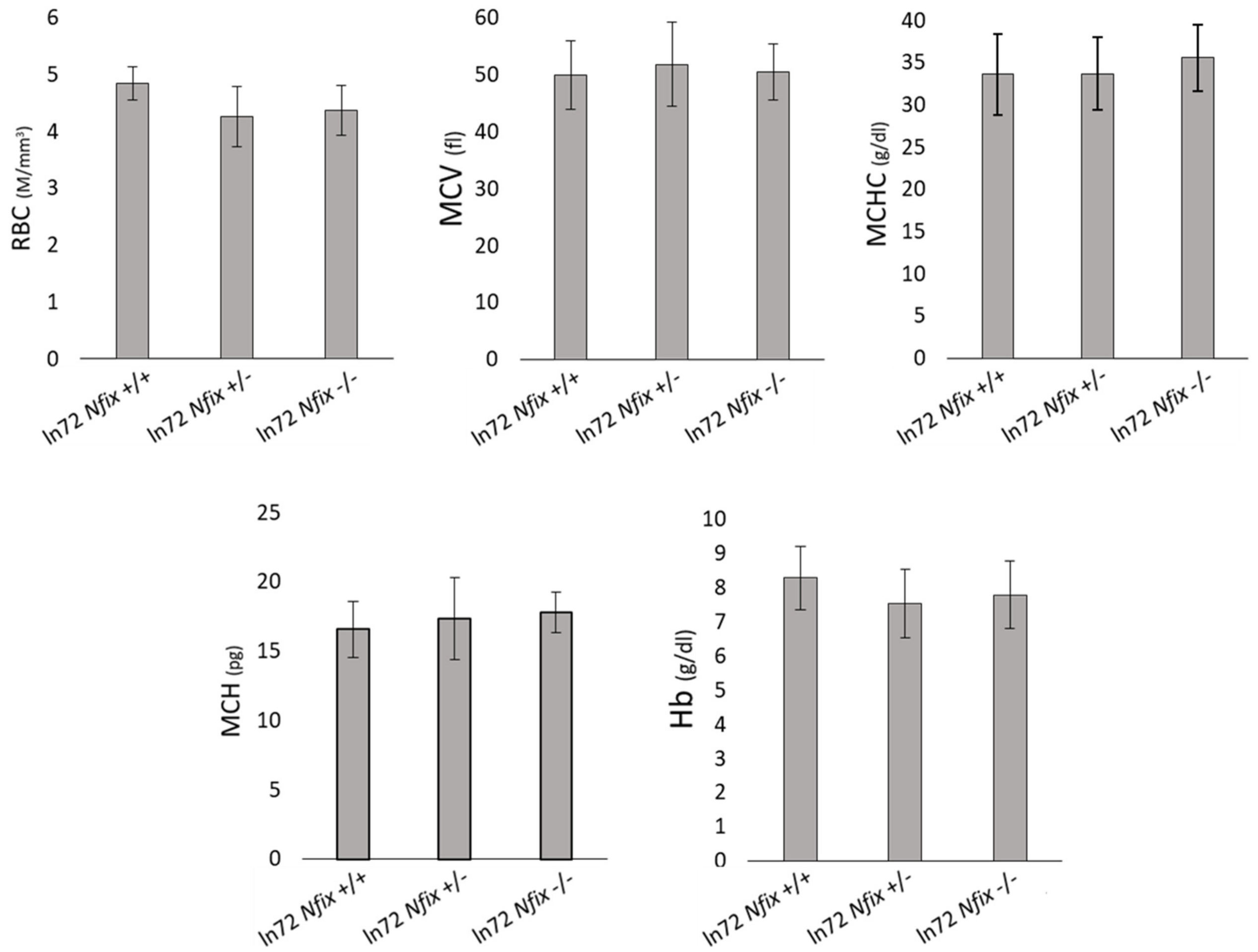

Figure S1. Hematological parameters from In72 *Nfix*+/+, In72 *Nfix*+/- and In72 *Nfix*-/- mice. Plots show values regarding RBC, MCV, MCHC, MCH and Hb parameters. The error bars represent the standard deviation from the mean.

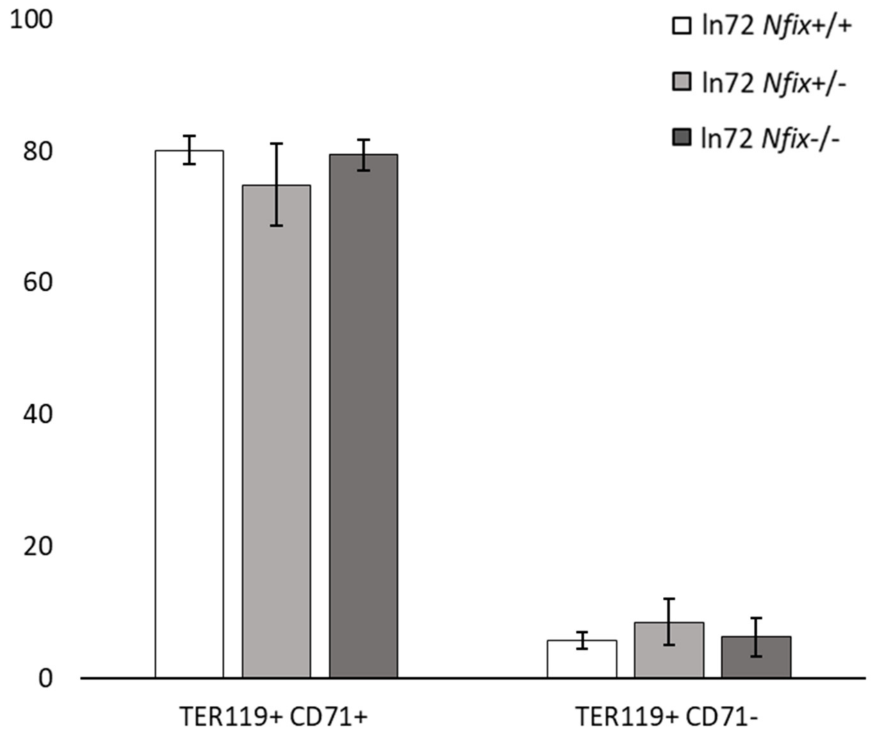

Figure S2. Flow cytometry from adult In72 *Nfix* mice. Bar plot represents erythropoiesis analysis according to Ter119/Cd71 levels of expression. The error bars represent the standard deviation from the mean.

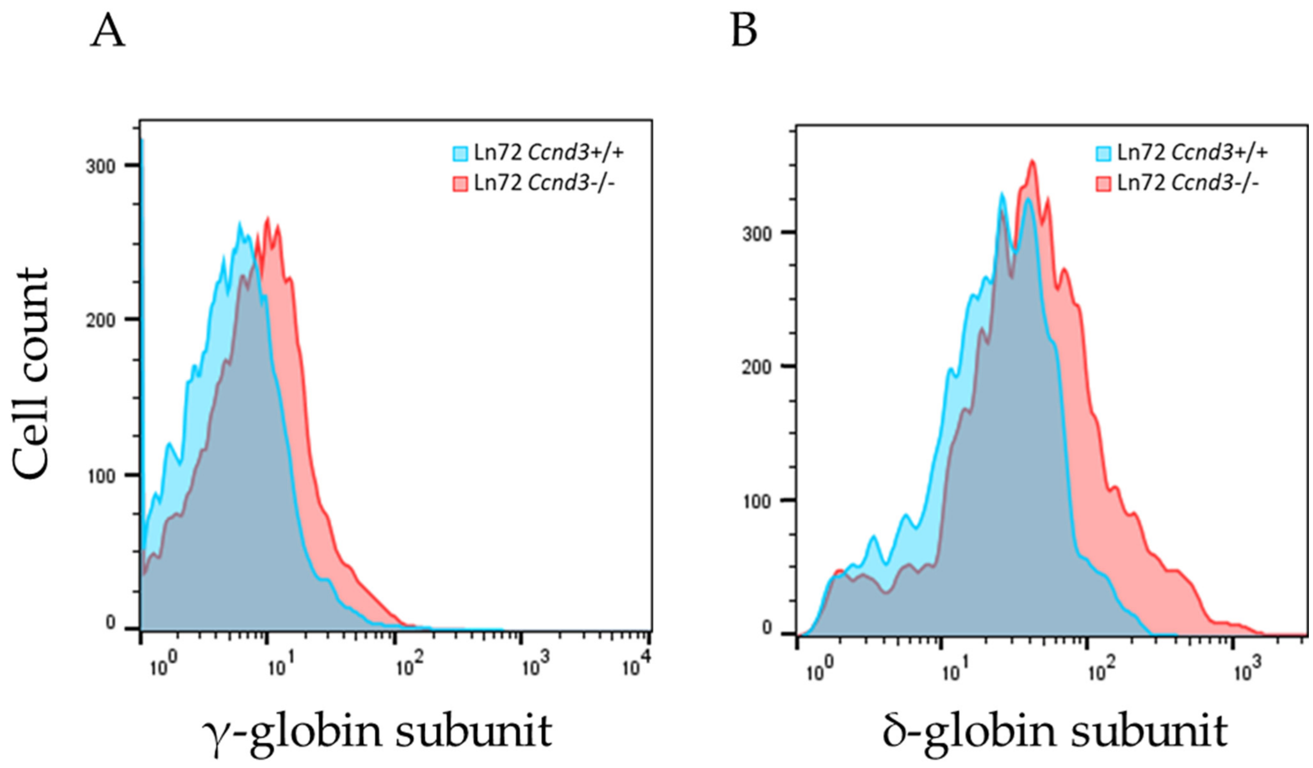

Figure S3. Histograms representing A) γ- and B) δ-globin subunits positive cells in ln72 *Ccnd3*+/+ and ln72 *Ccnd3*-/- mice models.

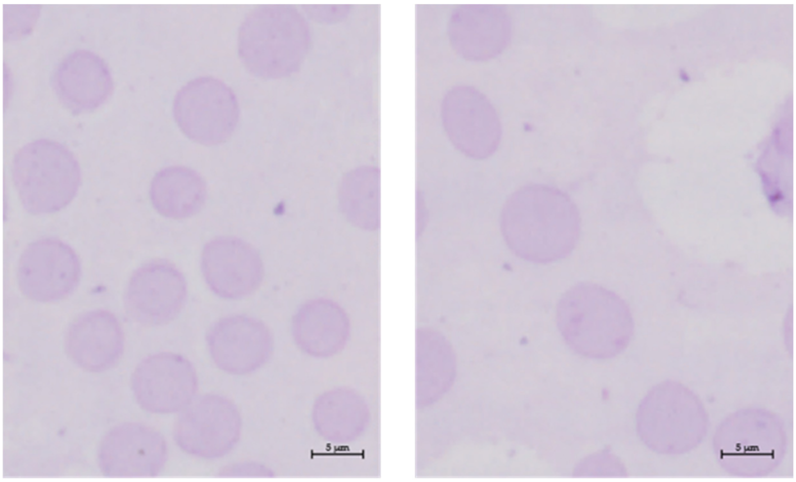

Ln72 Ccnd3+/+ Ln72 Ccnd3-/-

Figure S4. Comparison between erythrocytes volume of ln72 *Ccnd3*+/+ and ln72 *Ccnd3*-/- mice models, left panel represents erythrocytes from ln72 *Ccnd3*+/+ while right panel represents ln72 *Ccnd3*+/+ mice erythrocytes. Bar scale= 5 µm

Supplementary materials and methods

Flow cytometry

Analyses were conducted on freshly isolated cells ($1x10^5$ each sample) from adult bone marrow, from ln72 *Ccnd3*+/+ and ln72 *Ccnd3*-/-. Before labeling, cells were fixed and permeabilized as described in the main text (materials and methods, 2.4). Labeling of the cells was performed by using anti-HBG (sc-21756; Santa Cruz Biotechnology, INC) or anti-HBD (LS-C718187; LSBio, LifeSpan Bioscience) antibodies at final concentration 1:50. Cells were incubated for 20 min at 4°C (dark room), washed in phosphate-buffered saline (5% bovine serum albumin), and re-suspended in fluorescence-activated cell sorting (FACS) flow solution (BD-Bioscience). Data was recorded through FACSCanto cytometer (BD Bioscience) and analyzed by FACSDiva software Version 6.1.3 (BD Bioscience) and Flowjo v7.6.5 (BD-Bioscience).

Blood smear staining

Blood smears were obtained from ln72 *Ccnd3*+/+ and ln72 *Ccnd3*-/- mice models and stained with RAL 555 KIT (CELLAVISION, RAL Diagnostics). Images were collected using Zeiss Axio Scan.Z1 (ZEISS).

Table S1. Gene name, sequence, and application of primers used in this study.

GENE	SPECIES	APPLICATION	PRIMER SEQUENCE (5'→3')
Ln72	Human	Genotyping	TAAGCCAGTGCCAGAAGAGC<br>TGATACCAACCTGCCCAGG
Ccnd3	Mouse	Genotyping	TCCATCCTGCGATGGCTCAC<br>TGCTGTCCATCTGCACGAGA<br>GAACGTTGTGACGTAGGAGC
Nfix	Mouse	Genotyping	ATGGACATGTCATGGGTGCGACAG<br>AACCAGAGGCACGAGAGCTTGTC<br>AAGCCCCTCAGCTCTAGCACAGAG
α-globin	Mouse	RT-qPCR	CACCACCCTGCCGATTTC<br>CTCACAGAGGCAAGGAATTTGTC
α-globin	Human	RT-qPCR	GCACGCTGGCGAGTATGG<br>TCGAAGTGCGGGAAGTAGGT
β-globin	Human	RT-qPCR	TTGGACCCAGAGGTTCTTTGA<br>TCACTAAAGGCACCGAGCACT
γ-globin	Human	RT-qPCR	CTGAGTGAACTGCACTGTGACAAG<br>TCTTTGCCGAAATGGATTGC
δ-globin	Human	RT-qPCR	AGGTGCTAGGTGCCTTTAGTGA<br>GGGTGAATTCCTTGCCAAAGTTGC
Klf1	Mouse	RT-qPCR	GGGAAGAGCTACACCAAGAGC<br>GTCCCAGGAGCAGGCATAAGG
KLF1	Human	RT-qPCR	CGGACACACAGGATGACTTCC<br>CCATGTCCTGCGCCTCTT
KLF1 prom	human	Cloning	ATCAAGATCTCCTCCAACGTCTGGGGTGTCTG<br>ATATTGCAAGGCTGGCTGGTGCCCACCCTG
NFIX	Human	Cloning	ATGAATTCATGTACTCCCCGTACTGCCTCACCCA<br>TATCTAGAATCAGAGGAACCAGGACTGAGACTG

Table S2. Data from RT-qPCR on ln72 *Ccnd3* mice

	Time point	Genotype	Average	St. deviation	T test
γ-globin	12.5dpc	Ln72 Ccnd3+/+	1		vs -/- n.s.
		Ln72 Ccnd3+/-	1.271126255	±0.018	vs -/- n.s.
		Ln72 Ccnd3-/-	1.538136281	± 0.008	
	14.5dpc	Ln72 Ccnd3+/+	1		vs -/- *
		Ln72 Ccnd3+/-	1.547564994	±0.013	vs -/- **
		Ln72 Ccnd3-/-	2.648177821	± 0.015	
	16.5dpc	Ln72 Ccnd3+/+	1		vs -/- *
		Ln72 Ccnd3+/-	1.208597056	± 0.35	vs -/- *
		Ln72 Ccnd3-/-	3.689264774	± 0.11	
	adult	Ln72 Ccnd3+/+	1		vs -/- ***
		Ln72 Ccnd3+/-	1.822145086	± 0.23	vs -/- ***
		Ln72 Ccnd3-/-	24.33055614	± 5.53	
δ-globin	12.5dpc	Ln72 Ccnd3+/+	1		vs -/- n.s.
		Ln72 Ccnd3+/-	1.015544976	± 0.25	vs -/- n.s.
		Ln72 Ccnd3-/-	1.153664217	±0.11	
	14.5dpc	Ln72 Ccnd3+/+	1		vs -/- *
		Ln72 Ccnd3+/-	1.154018752	± 0.15	vs -/- **
		Ln72 Ccnd3-/-	1.63202897	± 0.18	
	16.5dpc	Ln72 Ccnd3+/+	1		vs -/-
		Ln72 Ccnd3+/-	1.3568	±0.2	vs -/-
		Ln72 Ccnd3-/-	2.06847652	± 0.36	
	adult	Ln72 Ccnd3+/+	1		vs -/- *
		Ln72 Ccnd3+/-	1.559598254	±0.21	vs -/- **
		Ln72 Ccnd3-/-	3.327866554	± 0.6	
β-globin	12.5dpc	Ln72 Ccnd3+/+	1		vs -/- n.s.
		Ln72 Ccnd3+/-	1.168	±0.085	vs -/- n.s.
		Ln72 Ccnd3-/-	1.122	±0.094	
	14.5dpc	Ln72 Ccnd3+/+	1		vs -/- n.s.
		Ln72 Ccnd3+/-	1.097	±0.178	vs -/- n.s.
		Ln72 Ccnd3-/-	1.137	±0.205	
	16.5dpc	Ln72 Ccnd3+/+	1		vs -/- n.s.
		Ln72 Ccnd3+/-	0.986976824	±0.176	vs -/- n.s.
		Ln72 Ccnd3-/-	1.133298677	± 0.2	
	adult	Ln72 Ccnd3+/+	1		vs -/- n.s.
		Ln72 Ccnd3+/-	1.024485296	±0.14	vs -/- n.s.
		Ln72 Ccnd3-/-	1.109896091	± 0.11	

Abbreviations: n.s. not significant. Asterisks indicate statistical significance (* P<0.05 ; ** P<0.01; *** P<0.001)

Table S3. Data from RT-qPCR on ln72 *Nfix* mice

	Time point	*Genotype*	*Average*	*St. deviation*	*T test*
γ-globin	12.5dpc	Ln72 Nfix+/+	1		vs -/- n.s.
		Ln72 Nfix +/-	1,0154728	±0.12	vs -/- n.s.
		Ln72 Nfix -/-	1,1225096	± 0.15	
	14.5dpc	Ln72 Nfix +/+	1		vs -/- n.s.
		Ln72 Nfix +/-	0,9866337	±0.19	vs -/- n.s.
		Ln72 Nfix -/-	1,0973192	± 0.14	
	16.5dpc	Ln72 Nfix +/+	1		vs -/- *
		Ln72 Nfix +/-	0,9611614	± 0.19	vs -/- *
		Ln72 Nfix -/-	1,4159609	± 0.33	
	14 dpb	Ln72 Nfix +/+	1		vs -/- **
		Ln72 Nfix +/-	1,2624509	± 0.25	vs -/-n.s.
		Ln72 Nfix -/-	1,7616441	± 0.24	
β-globin	12.5dpc	Ln72 Nfix +/+	1		vs -/- n.s.
		Ln72 Nfix +/-	1,0747441	±0,29	vs -/- n.s.
		Ln72 Nfix -/-	1,108689	±0,24	
	14.5dpc	Ln72 Nfix +/+	1		vs -/- n.s
		Ln72 Nfix +/-	0,8329028	±0,109	vs -/-n.s.
		Ln72 Nfix -/-	1,0782588	± 0.18	
	16.5dpc	Ln72 Nfix +/+	1		vs -/-n.s.
		Ln72 Nfix +/-	0,8479486	±0.26	vs -/-n.s.
		Ln72 Nfix -/-	0,9486068	± 0.10	
	14 dpb	Ln72 Nfix +/+	1		vs -/-n.s.
		Ln72 Nfix +/-	0,7948218	±0.21	vs -/-n.s.
		Ln72 Nfix -/-	1,0325262	±0,09	

Abbreviations: n.s. not significant. Asterisks indicate statistical significance (* $P<0.05$; ** $P<0.01$; *** $P<0.001$)

Table S4. Hematological parameters of In72 Ccnd3 mice.

		**In72 Ccnd3 +/+**	**In72 Ccnd3 +/-**	**In72 Ccnd3 -/-**
RBC (M/mm3)	Value	8.95	8.41	6.57
	st.dev	±0.3	±1.3	±0.47
	test t	vs -/- **	vs -/- n.s	
Hb g/dl	Value	13.96	12.7	12.53
	st.dev	±0.9	±2.15	±0.21
	test t	vs -/- n.s	vs -/- n.s	
MCV fl	Value	45.06	48.5	64.5
	st.dev	±2.93	±0.75	±1.25
	test t	vs -/- ***	vs -/- n.s	
MCH pg	Value	15.52	15.03	19.1
	st.dev	±0.8	±0.32	±1.73
	test t	vs -/-*	vs -/- n.s	
MCHC g/dl	Value	34.74	31.1	29.67
	st.dev	±3.73	±0.44	±2.07
	test t	vs -/-**	vs -/- n.s	

Abbreviations: n.s. not significant. Asterisks indicate statistical significance (* $P<0.05$; ** $P<0.01$; *** $P<0.001$)

Table S5. Hematological parameters of In72 Nfix mice.

		In72 Nfix +/+	In72 Nfix +/-	In72 Nfix -/-
RBC (M/mm3)	Value	4.81	4.67	4.54
	st.dev	±0.38	±1.35	±0.5
	test t	vs -/- n.s	vs -/- n.s	
Hb g/dl	Value	8.28	7.53	7.79
	st.dev	±0.92	±1	±1
	test t	vs -/- n.s	vs -/- n.s	
MCV fl	Value	49.93	51.83	50.54
	st.dev	±6	±7.36	±4.89
	test t	vs -/- n.s	vs -/- n.s	
MCH pg	Value	16.58	17.34	17.79
	st.dev	±2.01	±2.94	±1.45
	test t	vs -/-n.s.	vs -/- n.s	
MCHC g/dl	Value	33.6	33.68	35.56
	st.dev	±4.78	±4.32	±3.97
	test t	vs -/- n.s	vs -/- n.s	

Abbreviations: n.s. not significant. Asterisks indicate statistical significance (* P<0.05 ; ** P<0.01; *** P<0.001)
